# Protective potential of hydroxysafflor yellow A in cerebral ischemia and reperfusion injury: An overview of evidence from experimental studies

**DOI:** 10.3389/fphar.2022.1063035

**Published:** 2022-12-15

**Authors:** Lu Yu, Zhe Jin, Mincheng Li, Huifang Liu, Jie Tao, Chuan Xu, Liwei Wang, Qiujuan Zhang

**Affiliations:** ^1^ Comprehensive Department of Traditional Chinese Medicine, First Department of Integration, Department of Neurology, Putuo Hospital, Shanghai University of Traditional Chinese Medicine, Shanghai, China; ^2^ Department of Neurology, Renji Hospital Baoshan Branch, School of Medicine, Shanghai Jiao Tong University, Shanghai, China; ^3^ Department of Neurology, Shanghai Jinshan Hospital of Integrated Traditional Chinese and Western Medicine, Shanghai, China; ^4^ Department of Neurology, Yueyang Hospital of Integrated Traditional Chinese and Western Medicine, Shanghai University of Traditional Chinese Medicine, Shanghai, China

**Keywords:** hydroxysafflor yellow A, cerebral ischemia-reperfusion injury, natural bioactive compounds, neuroprotection, pathophysiological mechanism, review

## Abstract

Ischemic stroke, mostly caused by thromboembolic or thrombotic arterial occlusions, is a primary leading cause of death worldwide with high morbidity and disability. Unfortunately, no specific medicine is available for the treatment of cerebral I/R injury due to its limitation of therapeutic window. Hydroxysafflor yellow A, a natural product extracted from *Carthamus tinctorius*, has been extensively investigated on its pharmacological properties in cerebrovascular diseases. However, review focusing on the beneficial role of HSYA against cerebral I/R injury is still lacking. In this paper, we reviewed the neuroprotective effect of HSYA in preclinical studies and the underlying mechanisms involved, as well as clinical data that support the pharmacological activities. Additionally, the sources, physicochemical properties, biosynthesis, safety and limitations of HSYA were also reviewed. As a result, HSYA possesses a wide range of beneficial effects against cerebral I/R injury, and its action mechanisms include anti-excitotoxicity, anti-oxidant stress, anti-apoptosis, anti-inflammation, attenuating BBB leakage and regulating autophagy. Collectively, HSYA might be applied as one of the promising alternatives in ischemic stroke treatment.

## 1 Introduction

Ischemic stroke, which accounts for more than 80% of all strokes, mostly induced by thromboembolic or thrombotic arterial occlusions, is a primary leading cause of death with high morbidity and disability worldwide in current years (Prabhakaran et al., 2015; Katan and Luft, 2018). More than half of the stroke victims remain with neurological deficits including numbness, hemiplegia, balance problems, loss of sensory, decreased reflexes, visual field defects, apraxia, and aphasia which require prolonged rehabilitation (Tsuchiya et al., 1992; Assayag et al., 2012). These neurological deficits are mainly attributed to cerebral ischemia and reperfusion (I/R) injury, which is characterized by a series of pathological events, including inflammatory response (Linnerbauer et al., 2020), oxidative stress (Liu et al., 2020), glutamate toxicity (Dohmen et al., 2005), energy metabolism disorders (Gong et al., 2018), apoptosis (Wen et al., 2019), and many other factors. Since the prevalence rate continues to increase, and the affected population has represented a younger trend, the prevention and treatment of stroke is the significant problem in China (Fu et al., 2020). Recently, treatment options for this stubborn disease are still limited due to the narrow therapeutic window. The recombinant tissue-type plasminogen activators (rtPAs) are nowadays the standard therapeutics in acute ischemic stroke, however the time to initiate intravenous thrombolysis is generally limited to within 4.5 h after the onset of symptoms (Ma et al., 2019).

**FIGURE 1:**
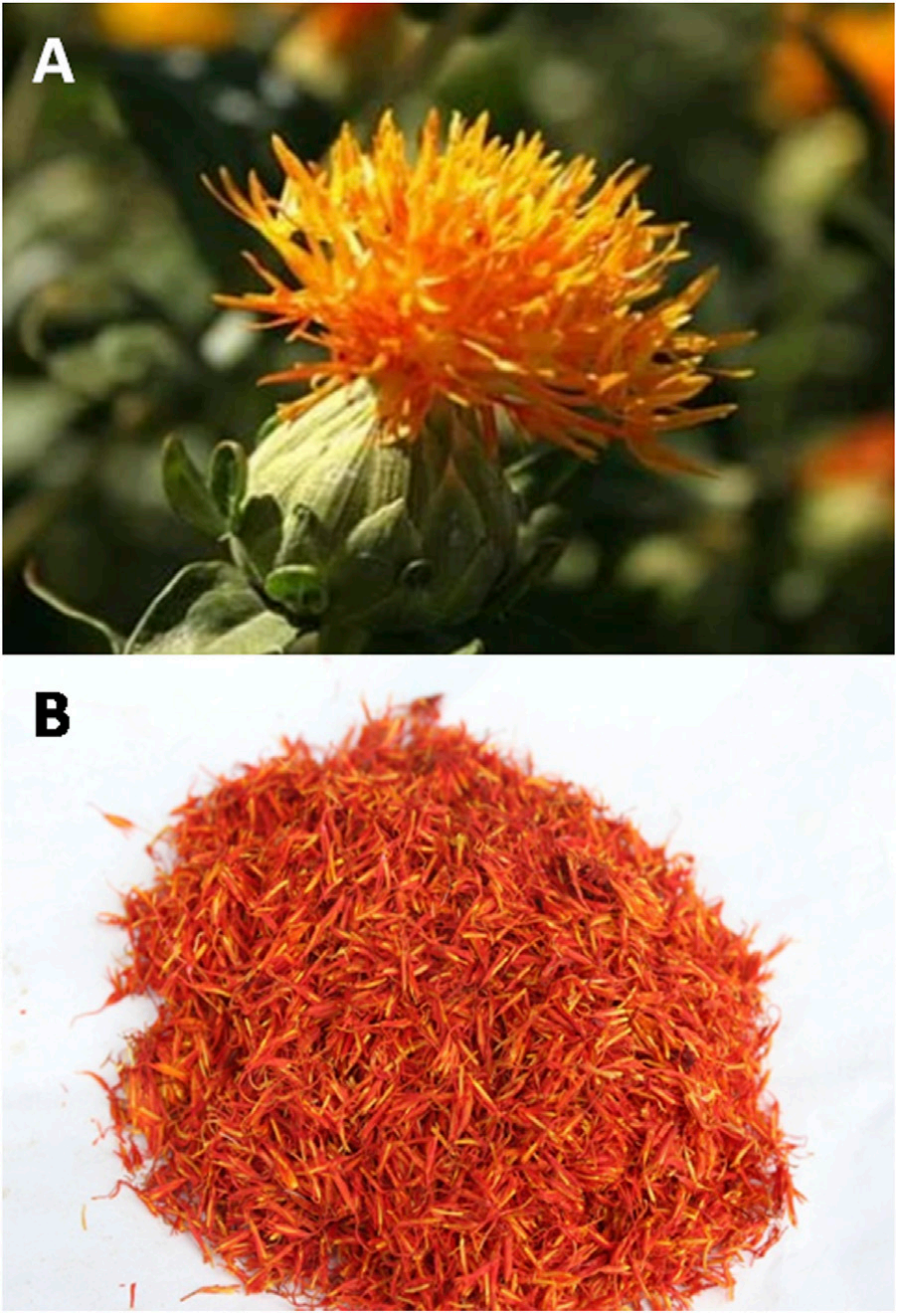
**(A)** *Cathamus tinctorius L*., **(B)** CarthamiFlos (the dried florets of *C. tinctorius*).

Based on the theories of traditional Chinese medicine, blood stasis syndrome is thought to be a common clinical syndrome type of ischemic stroke which requires activating blood and resolving stasis method (Wang et al., 2020). The treatment of cardio-cerebra-vascular ischemic diseases by natural medicine is featured by high efficacy and low adverse effects and has a long history with a unique theoretical system (Gu et al., 2014; Chen et al., 2017). *Carthamus tinctorius* L. is a branching, thistle-like herbaceous annual plant (Figure 1A). The dried florets of *Carthamus tinctorius* (Figure 1B) has been widely applied in the treatment of cerebrovascular disease for thousands of years (Yu et al., 2018), which is described in the Compendium of Materia Medica as being able to “invigorate the circulation of blood”, suggesting its positive role in the circulation system (Xu et al., 2012).

Hydroxysafflor yellow A (HSYA) is a major bioactive component first isolated from *Carthami flos*, the flower of *Carthamus tinctorius* L. in 1993 (Yue et al., 2013), and has been used in the clinical treatment for ischemci cerebrovascular disease. Emerging evidences have revealed that HSYA possesses a wide range of biological activities against I/R injury, such as anti-oxidative, anti-inflammatory, anti-apoptotic effects, relieving BBB damage (Xu et al., 2021), reducing cerebral infarction, attenuating cerebral edema, promoting neurological recovery and improving cognitive function (Yu et al., 2018; Yu et al., 2020a). Thus, HSYA is expected to be developed as a promising candidate for combating ischemic stroke. Up to now, several reviews on traditional Chinese herb have been conducted, in which the therapeutic effects of HSYA on cardio-cerebrovascular diseases were mentioned. However, the pharmacological properties of HSYA against cerebral I/R injury has not been comprehensively reviewed. In this article, we focused on the neuropharmacological properties of HSYA, the therapeutic effects of HSYA in preclinical models of ischemic stroke and the underlying mechanisms involved, as well as clinical data that support its neuroprotective action. Moreover, the sources, physicochemical properties, biosynthesis, and safety of HSYA were also reviewed here.

## 2 Sources of HSYA

Safflower (*Carthamus tinctorius* L.), as the natural source of HSYA, is widely planted worldwide. In China, it is also cultivated with planting area of about 30,000 hm^2^–58,000 hm^2^ and Xinjiang province is the major safflower production area providing more than 80% dried flowers and seeds (Zhao et al., 2020). *Carthami flos*, the dried flower of safflower, is a classic medicine for promoting blood circulation and removing blood stasis. Geographical origins, color and harvest time are the main factors influencing the content of HSYA containing in safflower. For example, HSYA cultivars in China are higher than that in Turkey, India and Kenya. The content of HSYA is higher in safflower with darker colors (Xu et al., 2018). And the most appropriate time to pick safflower is the morning of the third or fourth day after the onset of flowering (Tian et al., 2007).

## 3 Physicochemical properties and biosynthesis

**FIGURE 2:**
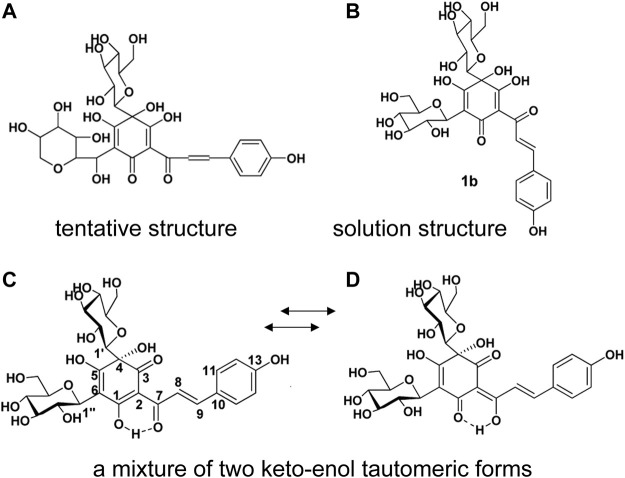
The structure of HSYA. **(A)** The tentative structure identified by Onodera et al., 1981, **(B)** the structure identified by Meselhy et al., **(C, D)** the structure identified by Feng et al., 2013.

It is well known that safflower yellow is the main active components in *Carthami flos* extract, including safflower yellow A, safflower yellow B, hydroxysafflow yellow A (HSYA), etc., (Zhang et al., 2016). Among these components, HSYA is the major bioactive component of *Carthami flos*, accounting for 85% of safflower yellow (Su et al., 2018). HSYA has a stable structure at pH 3-7 and below 60°C. However, it is easily degraded by light, high temperature, strong acidic and alkaline conditions (Fan et al., 2011b). In 1981, a quinochalcone C-glycoside, named safflomin A, was firstly isolated from *C. tinctorius* by Onodera et al. (1981). Since its ^1^H and ^13^C NMR data and other related information were aligned with that of HSYA, the tentative structure Figure 2A was proposed. In 1993, Meselhy et al. (1993) isolated the compound from *C. tinctorius* and described it as a new quinochalcone C-glycoside. Meanwhile, its structure was identified as Figure 2B and was formally named hydroxysafflor yellow A. In 2013, Feng et al. put forward that HSYA was a mixture of two keto-enol tautomeric forms (Figures 2C,D), with the 1-enol-3,7-diketo form, is the preferred tautomer (Feng et al., 2013) which amended the conclusion that HSYA belongs to “quinochalcone-C-glycoside structures”. Nowadays, HSYA is mainly obtained from plants at about 1%–3%. Planting environment, harvesting time and anthropogenic induction are the significant factors to increase the yield of HSYA. Additionally, HSYA is produced mainly through the phenylalanine metabolic pathway, of which chalcone synthase genes (CHSs) are the rate-limiting enzymes (Xue et al., 2021). CHSs expression and HSYA accumulation are peaked after 3–4 days of flowering (Kang, 2014). It’s worth noting that enzymatic catalysis is efficient to promote biosynthesis of natural products. Methyl jasmonate (MeJA), a well-known exogenous inducing factor, has been reported to promote the biosynthesis of HSYA through regulating the expression of upstream and downstream genes in the flavonoid biosynthesis pathway, such as CHSs, CHIs, F3Ms, ANRs etc (Chen et al., 2020).

## 4 Protective effects and mechanisms

**FIGURE 3:**
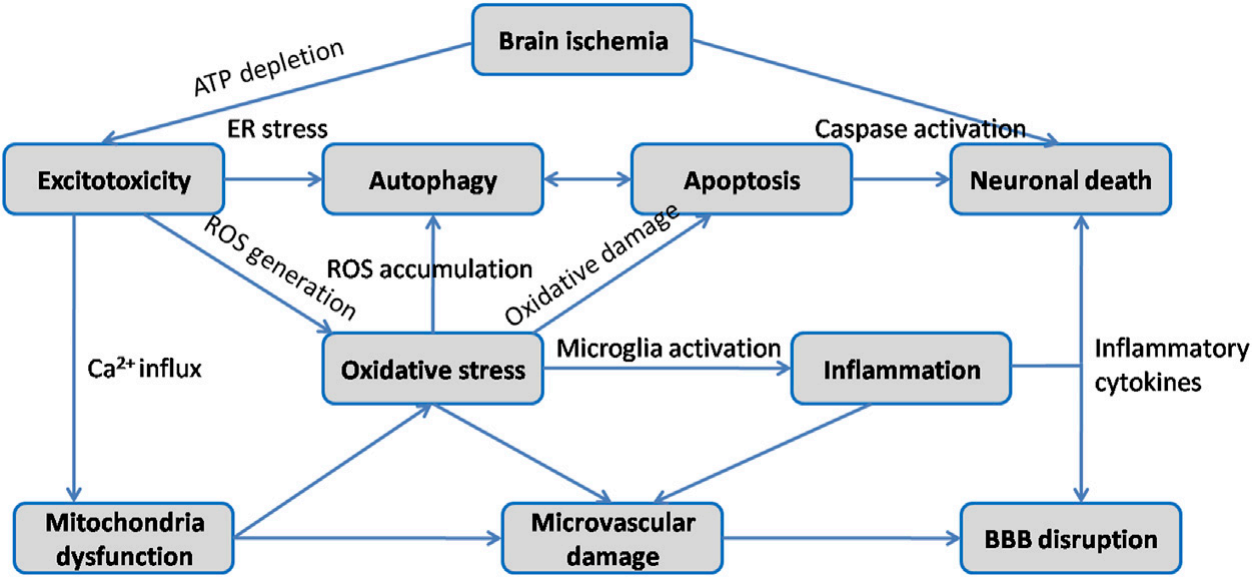
Schematic diagram of the main pathophysiological mechanisms in cerebral I/R injury.

Ischemic stroke occurs when the blockage of brain artery causes a reduction of regional cerebral blood flow, resulting in deleterious effects on neurons followed by a series of pathological processes such as excitotoxicity, oxidative stress, inflammatory response, neuronal apoptosis, BBB disruption and autophagy (Figure 3). Since extensive work aims to explore neuroprotective therapeutics for stroke, multiple animal models have been developed to reproduce both focal and global ischemic stroke. Middle cerebral artery occlusion (MCAO) model is the most widely used experimental model for inducing focal cerebral ischemia in rodents, while four vessel occlusion and two vessel occlusion methods are commonly used in global cerebral ischemia (Bacigaluppi et al., 2010). Various studies have indicated that HSYA dose-dependently improves neurological deficit scores, reduces cerebral infarct volume, attenuates brain edema and recovers cognitive impairment (Zhu et al., 2005; Yu et al., 2018; Yu et al., 2020a). The initial mechanisms are closely associated with inhibitory effect of HSYA on thrombosis formation and platelet aggregation following focal cerebral ischemia. And HSYA could improve blood rheological parameters as well (Zhu et al., 2005). In contrast, it is reported that HSYA administration has no impact on cerebral blood flow, blood pressure and heart rate in Beagle dogs (Sun et al., 2018). The discrepancies would be explained by differences in animal model. *In vitro* studies revealed that HSYA protected brain microvascular endothelial cells (BMECs) injury induced by oxygen and glucose deprivation/reoxygenation (OGD/R) *via* inhibiting autophagy, which was associated with its regulation of the Class I PI3K/AKT/mTOR pathway (Yang et al., 2018). Moreover, HSYA exhibits protective action on neuronal damage following glutamate and sodium cyanide (NaCN) exposure in fetal cortical cells (Zhu et al., 2003). These results indicate that HSYA is a promising therapeutic agent for cerebral I/R injury treatment. To further support the its positive role in ischemic stroke, we review the underlying mechanisms which are correlated with the activities of HSYA to inhibit excitotoxicity, oxidative stress, inflammation, apoptosis, BBB damage, as well as regulate autophagy on below.

### 4.1 Inhibiting excitotoxicity

Excitotoxicity is a primary stage of neuronal injury following cerebral ischemia. It is triggered by neuronal stimulation with high concentration of glutamate and overactivation of glutamate receptors (Wang et al., 2018). N-methyl-d-aspartate (NMDA) subtype of glutamate receptors plays an important role in mediating glutamate accumulation at synapses, which is caused by high permeability of calcium (Lai et al., 2014). Overactivation of NMDA receptors (NMDARs) containing the NR2A and NR2B subunits is the pivotal reason in glutamate-provoked excitotoxic neuronal damage (Yang et al., 2010). Yang et al. (2010) conducted an *in vitro* study on NMDA-induced injury in rat primary neurons to investigate the effect of HSYA on NMDAR-mediated neurotoxicity. HSYA was claimed to attenuate the excitotoxic neuronal death, meanwhile over-expression of NR2B subtype by NMDA stimuli was reversed by HSYA, which indicated the neuroprotection of HSYA against NMDA-induced neuronal glutamate excitotoxicity. Since an excessive glutamate release triggers excitotoxic damage through the overactivation of NMDARs following brain ischemia (Soriaet al., 2014), Wang et al. further investigated the related mechanism of HSYA’s protective effect against glutamatergic excitotoxicity in NMDA-mediated and OGD-induced neuronal injury (Wang et al., 2016). HSYA was observed to inhibit postsynaptic NMDAR activity and NMDAR-mediated neuronal membrane depolarization under oxygen and glucose deprivation circumstance. Meanwhile, it was further confirmed to suppress pre-synaptic glutamate transmitter release (Wang et al., 2016). Moreover, through NMDAR-dependent manner, intracellular rapid influx of calcium initiated by glutamate release has been verified to be responsible for neuronal excitotoxicity (Tehse and Taghibiglou, 2019). Apart from that, HSYA was demonstrated to inhibit the increase of NMDAR-mediated Ca^2+^ concentration and NMDAR-dependent ischemic long-term potentiation (LTP) induced by OGD for protecting hippocampal neurons from excitotoxic damage (Wang et al., 2016). Taken together, these studies indicate that HSYA could ameliorate neuronal excitotoxicity after cerebral I/R injury *via* suppressing the overactivation of NMDARs, and consequently inhibiting excessive neurotransmitter release, neuronal membrane depolarization, overload of calcium and ischemic LTP, which are mediated by or depend on NMDAR.

### 4.2 Ameliorating oxidative stress

It is well known that anti-free radical system of nervous tissues is relatively weaker than other organs in the human body, which means that neurons are more prone to oxidative damage than other tissues (Xie et al., 2018). Under cerebral ischemic conditions, several pathological mechanisms, including neuronal excitotoxicity, excessive Ca^2+^ influx, mitochondrial dysfunction, may cause free radical damage (Yu et al., 2020b). In rat brain mitochondria, Tian et al. (2008) revealed that HSYA could inhibit Ca^2+^- and H_2_O_2_-induced swelling of mitochondria and generation of ROS, enhance ATP levels and improve mitochondrial energy metabolism. In PC12 cells and primary hippocampal neurons, Fan and Fangma et al. provided the *in vitro* evidence that HSYA could attenuate neuronal damage *via* reversing the decrease of superoxide dismutase (SOD) and glutathioneperoxidase (GSH-Px) activity, suppressing the increase of reactive oxygen species (ROS) and malondialdehyde (MDA) levels after OGD/R-induced injury (Wei et al., 2005; Fan et al., 2011a; Fangma et al., 2021). Since the release of cytochrome c from mitochondria has been evidenced to be mediated by ROS (Chung et al., 2021), HSYA was further demonstrated to significantly decrease the cytochrome c in the cytosol (Fan et al., 2011a). The exact mechanisms underlying the antioxidant effects of HSYA remain unclear. Silent information regulator 1 (SIRT1), a deacetylase, is involved in the regulation of cell survival, energy metabolism, anti-apoptosis (Ding et al., 2017). It has been proved to exert a positive role in cerebral ischemic injury (Ding et al., 2017). In MCAO rats and OGD/R-injured primary neurons, Fangma et al. (2021) provided the evidence that HSYA regulated the SIRT1 pathway. However, the effect of HSYA on SIRT1 was restrained with SIRT1-specific inhibitor EX527, suggesting the pivotal role of SIRT1 in neuroprotection of HSYA. Ferroptosis and parthanatos are two types of programmed cell death associated with cerebral ischemia. Excessive ROS may stimulate cell death pathway and trigger a series inflammation reaction (Tang et al., 2019). In OGD/R-insulted PC12 cells, Chen et al. (2022) found that HSYA limited ferroptosis and parthanatos to alleviate oxidative stress through suppressing PARP-1 overactivation and attenuating the production of excessive PAR polymer and translocation of AIF nuclear. Moreover, the excessive generation of ROS activates opening of mitochondrial permeability transition pore (mPTP) during I/R injury to further increase ROS production resulting in mitochondrial dysfunction, which is considered as a critical contributor to neuronal damage (Granger and Kvietys, 2015). In MCAO rats, Ramagiri et al. verified that HSYA could inhibit mPTP opening induced by oxidative stress (Ramagiri and Taliyan, 2016). HSYA was also proved to suppress the overexpression of 12/15-LOX, the enzyme involved in oxidative stress after MCAO (Sun et al., 2012). Collectively, these studies demonstrate that HSYA could mitigate oxidative stress evoked by I/R injury through increasing SOD and GSH-Px activity, inhibiting ROS and MDA levels, decreasing the cytochrome c in the cytosol, upregulating the SIRT1 pathway, suppressing mPTP opening, as well as limiting ferroptosis and parthanatos.

### 4.3 Anti-inflammation

Neuroinflammation has been recognized as a crucial pathological process following cerebral ischemia-reperfusion injury (Sun et al., 2020), which is characterized by the production of inflammatory cyto- and chemokines, as well as the infiltration of leukocyte into ischemic tissues (Franke et al., 2021). HSYA has been exhibited an anti-inflammatory role in both MCAO rats and OGD/R-injured neurons (Ye and Gao, 2008). In MCAO mice and LPS-treated microglia and neurons, HSYA was found to suppress the excessive secretion of inflammatory cytokines through inhibiting TLR4-mediated signaling pathway (Lv et al., 2015; Lv et al., 2016). In another study, HSYA was demonstrated to improve OGD/R-injured BV2 microglia viability by limiting pro-inflammatory cytokines (Li et al., 2013). Glycogen synthase kinase-3 (GSK-3) is a serine-threonine kinase composed of both alpha and beta isoforms (Eldar-Finkelman and Martinez, 2011), which has been evidenced to participate in the production of pro-inflammatory factors. Thus, inhibition of this kinase has been recognized as a molecular brake to limit inflammatory response (Cai et al., 2021). In MCAO rats, Yang et al. (2020) found that HSYA elevated GSK-3β phosphorylation levels and suppressed nuclear factor kappa B (NF-κB) activation in the ischemic penumbra, which manifested its anti-inflammatory properties by regulating GSK-3β. Glial fibrillary acidic protein (GFAP) is a crucial cytoskeletal component of astrocytes, as the contributor to trigger inflammatory response once excessively activated. Deng et al. revealed that HSYA attenuated inflammatory response through upregulating GFAP and reversing the increasing level of intercellular adhesion molecular 1(ICAM-1) in MCAO rats (Deng et al., 2018). Meanwhile, elevated inflammatory mediators, such as IL-1β, TNF-α and NF-κB were suppressed by HSYA (Deng et al., 2018). These results suggest that suppressing the TLR4-mediated pathway and TLR4-induced downstream effectors, increasing GSK-3β phosphorylation and GFAP expression contributed to the anti-inflammatory effects of HSYA following cerebral I/R injury.

### 4.4 Anti-apoptosis

Apoptosis is one of the two types of cell death produced by cerebral ischemia injury, which is triggered by either extrinsic or intrinsic stimuli (Radak et al., 2017). The intrinsic stimuli for apoptosis are *via* a series of mitochondrial signaling pathways (Yu et al., 2020b). Huang et al. found that HSYA increased viability of brain microvascular endothelial cells (BMECs) after OGD/R (Huang et al., 2021). Additionally, HSYA decreased the export of cytochrome c from mitochondrial by inhibiting mPTP opening *via* the regulation of MEK/ERK/CypD pathway in both OGD/R and MCAO models (Huang et al., 2021). Moreover, HSYA was identified to enhance mitochondrial function and biogenesis *via* inhibiting phenylalanine synthesis in OGD/R-injured primary neurons and PC12 cells (Chen et al., 2019). PH domain leucine-rich repeat protein phosphatase-1 (PHLPP1) has been found to participate in the regulation of cell survival and cell apoptosis (Aviv and Krishenbaum, 2010). PHLPP1 gene deletion could ameliorate cerebral ischemic injury implying its critical role in neuroprotection (Chen B. et al., 2013). In OGD/R-treated BMECs, HSYA was found to reverse the increased PHLPP1 evoked by OGD/R and its protective action was abolished once PHLPP1 knockout, which indicated that HSYA attenuated cellular apoptosis following ischemic-reperfusion injury in PHLPP1-dependent manner (Cao et al., 2020). A growing number of literatures demonstrated that the activation of PI3K/Akt pathway ameliorated I/R-induced apoptosis (Chang et al., 2018; Zhou et al., 2021). Moreover, glycogen synthase kinase 3β (GSK3β), an important downstream target of the Akt signaling, has been evidenced to regulate key steps involved in intrinsic apoptotic and extrinsic apoptotic pathways (Chen et al., 2014). In MCAO rats, Chen et al. (2013b) provided the consistent results that HSYA inhibited cellular apoptosis following I/R injury by increasing the phosphorylation levels of Akt and GSK3β. Furthermore, the application of wortmannin, an inhibitor of PI3K, revealed that PI3K/Akt pathway played a positive role in HSYA-mediated neuroprotective effect (Chen L. et al., 2013). Altogether, these studies demonstrate that HSYA could attenuate apoptosis after cerebral I/R injury by inhibiting mPTP open *via* MEK/ERK/CypD pathway, enhancing mitochondrial function and biogenesis, increasing PHLPP1 level, as well as regulating PI3K/Akt/GSK3β pathway.

### 4.5 Attenuating BBB damage

The blood-brain barrier (BBB), a unique anatomical and physiological interface between peripheral circulation and central nervous system (Daneman and Prat, 2015), regulates the trafficking of solutes, fluid and cells at blood-brain interface (Jiang et al., 2018). BBB integrity will be damaged under cerebral ischemia condition, leading to the development of brain injury and subsequent neurological impairment (Abdullahi et al., 2018). Tan et al. constructed an *in vitro* BBB model to verify that FDA-approved adenosine receptor agonist Lexiscan (Lex) promoted HSYA accumulation in the brain by transitory enhancement of BBB permeability. Accordingly, the combination of HSYA and Lex exhibited a better protective performance against I/R injury in MCAO rats than the single HSYA (Tan et al., 2020). Moreover, in MCAO rats, Sun et al. (2012) demonstrated that HSYA reduced the increase of serum IgG following brain ischemia by more than 50%, indicating the amelioration effect of HSYA against BBB disruption subjected to cerebral ischemia. Lv et al. used the integrated method of serial affinity chromatography and shotgun proteomics analysis to explore the underlying mechanism of HSYA’s protective effect on BBB damage in anti-inflammatory patterns in MCAO mice (Lv and Fu, 2018). The data showed that HSYA regulated the tight junction *via* TLR4/PI3K/AKT/JNK1/2/14-3-3ε/NF-κBp65 pathway and modulated BBB permeability *via* suppressing inflammation (Lv and Fu, 2018). Li et al. (2022) performed photothrombotic stroke model in C57BL/6J mice to imitate cerebral ischemia, in which HSYA was verified to protect ZO-1 stability, a tight junction protein, for reducing cerebral vascular leakage *via* blocking HIF-1α/NOX2 signaling cascades (Li et al., 2022). The findings demonstrated the significant role of HIF-1α in NOXs activation and the regulatory effect of HSYA on HIF-1α/NOX2 signaling cascades for protecting cerebral vessel integrity. Apart from that, the caveolin pathway has been found to play an important role in preserving and protecting BBB integrity (Huang et al., 2018). Caveolin-1 (Cav-1) could reduce BBB permeability destroyed by ischemic stroke through downregulating MMP9 (Huang et al., 2018). In OGD/R-injured BMECs, Cao et al. provided consistent results that HSYA exerted neuroprotective property by stimulating Cav-1 pathway, which validated the functioning of HSYA in rescuing BBB (Cao et al., 2020). These results indicate that HSYA may attenuate BBB leakage after I/R injury *via* regulating the tight junction, stimulating the caveolin-1 pathway and blocking HIF-1α/NOX2 signaling cascades.

### 4.6 Regulating autophagy

Autophagy is a cellular catabolic process that acts as a double-edged sword under pathological conditions, contributing to either cell survival or cell damage (Martinet et al., 2009). It is commonly agreed that autophagy is closely associated with heart disease, cancer and neurodegenerative disease (Martinet et al., 2009). In recent years, growing evidence has revealed that autophagy confers cytoprotection against various pathological stresses, including ischemia/reperfusion injury (Lai et al., 2020). In MCAO rats, Qi et al. (2014) found that HSYA promoted autophagy in the penumbra through activating AKT-dependent autophagy pathway, which was subsequently verified as a potential mechanism in the HSYA-mediated neuroprotection. In contrast, Yang et al. (2018); Zhang et al. (2022) observed that HSYA inhibited autophagy following ischemia for exerting neuroprotection in OGD/R-insulted BMECs and MCAO rats, which is inconsistent with the observation of Qi et al. A body of studies has demonstrated that autophagy increases in cerebral I/R injury, and both harmful and protective potentials of autophagy have been reported (Wang et al., 2013; Luo et al., 2014). Yang et al. (2018) found that HSYA suppressed excessive autophagy evoked by OGD/R injury in BMECs and such inhibition was partially associated with the activation of Class I PI3K/AKT/mTOR pathway. Similarly, Zhang et al. (2022) revealed that HSYA suppressed autophagy induced by brain ischemia probably through limiting the expressions of HIF-1, BNIP3 and Notch 1. All together, these results demonstrate that HSYA may regulate autophagy induced by cerebral I/R injury *via* activating AKT-related pathway and the Class I PI3K/AKT/mTOR pathway, as well as decreasing the expressions of HIF-1, BNIP3 and Notch 1.

**FIGURE 4:**
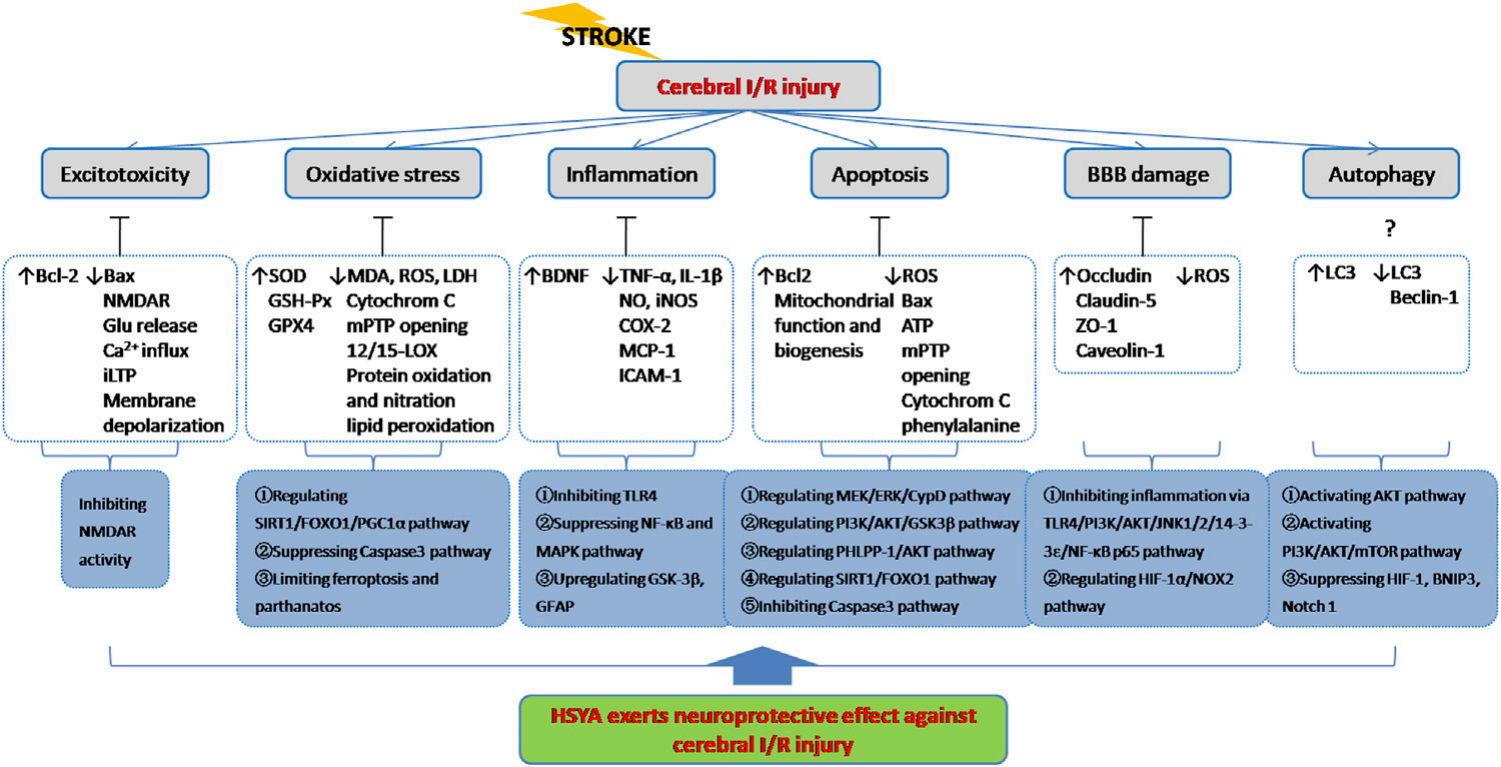
HSYA acts on the functional targets and signaling pathways of cerebral I/R injury. Indicator: ↑, upgrade; ↓, downgrade; ?, undetermined.

It is known that ischemic stroke involves several major pathogeneses including excitotoxicity, oxidative stress, inflammation, apoptosis and BBB damage etc. As described above, HSYA exerts potent neuroprotection against cerebral I/R injury through complex signaling pathways and exhibits a definite therapeutic effect for brain ischemia treatment **(** Figure 4 **)**.

## 5 Clinical application

Safflow yellow injection (SYI) contains 90% HSYA (45 mg HSYA per 50 mg SYI), and it has been reported to use clinically for ischemic stroke (Li L. J. et al., 2015). Li et al. (2015b) provided clinical evidence that SYI exerts beneficial effect for acute cerebral infarction. The study was a prospective, single-blinded, and randomized controlled trial and conducted in 108 patients after informed consent and screening. All patients were randomized to either SYI group or control group. SYI (80 mg) was given to the SYI group and placebo (0 mg) injection was given to the control group by intravenous drop once daily for 14 days. The results showed that SYI improved neurological deficits and hemorheological index, including red blood cell deformation and red blood cell aggregation. Prothrombin time was increased and fibrinogen, TNF-α, IL-1β and IL-6 were decreased in patients treated with SYI on day 14 after treatment (Li L. J. et al., 2015). Hu et al. conducted a multicenter, randomized, double-blind, multiple-dose and active-controlled clinical trial for assessing effect and safety of HSYA injection in 266 patients with acute ischemic stroke of blood stasis syndrome. 25 mg/d, 50 mg/d, and 70 mg/d HSYA injection were administrated by intravenous infusion for 14 consecutive days. Scores of NIHSS and BI at days 90 after treatment as well as improvement degree of blood stasis syndrome at days 30 and 60 after treatment in the medium- and high-dose HSYA groups were higher than the control group. Thus, HSYA injection was proved to be safe and well-tolerated at all doses for acute ischemic stroke patients with blood stasis syndrome (Hu et al., 2020). Although a growing number of preclinical studies have displayed the significant protective potential of HSYA against cerebral I/R injury, there is still a lack of convincing evidence with high methodological quality for the efficacy and safety of HSYA in acute cerebral infarction treatment.

## 6 Safety

Subchronic toxicity studies in SD rats signified a safety concern of HSYA (Liu et al., 2004). The exposure of HSYA at the doses of 180, 60 mg/kg by daily intraperitoneal injection for 90 days period caused a prolonged blood coagulation time. However, the normal blood coagulation process wasn’t influenced. No animal was observed to die from hemorrhaging. Kidney injury, including round tubular figures and a breaking-off of the tubular epithelium in histological slices, was observed in the rats with 180 mg/kg HSYA. Although the liver index was increased with 180 mg/kg HSYA, no pathological change of liver histiocytes has been found. The data indicated that the great amount of HSYA is excreted by kidney and induces a slight nephrotoxicity (Liu et al., 2004). On the other hand, some factors in drug metabolism will cause unsafe events. In a clinical experiment of 36 Chinese healthy adults, single doses (25 mg, 50 mg, and 75 mg) of injectable powder of pure HSYA (IPPH) displayed moderate linear pharmacokinetic properties. And seven successive days’ administration of IPPH didn’t cause the *in vivo* drug accumulation, but leaded to the decrease of its system exposure and prolonging of the drug elimination time (Li C. Y. et al., 2015). It is important to note that HSYA can either inhibit or induce activities of CYP1A2, CYP2C11 and CYP3A1, which may be correlated with the significant changes of maximum plasma concentration (C_max_) and area under the plasma concentration versus time curve (AUC) after multiple drug administration. Thus, co-administration of some CYP substrates with HSYA may need dose adjustment to avoid some herb-drug interaction (Xu et al., 2014). Besides, the C_max_ and AUC of female was generally larger than that of male, which may be influenced by sex differences in body weight, proportions of muscular and adipose tissues, gastrointestinal and renal blood flows, drug enzyme activity and hormonal factors (Li et al., 2011). Therefore, in the clinical application of HSYA, the drug dosage, the combination of drugs and the physical condition of patients should be fully considered to avoid adverse reaction.

## 7 Limitations and further perspectives

Although HSYA might be applied as one of the promising alternatives in ischemic stroke treatment, there still remain some limitations. Firstly, HSYA is easily degraded in the process of storage, extraction and separation procedure due to the chemical instability. Secondly, although oral administration is of great significance among many administration routes because of its convenience and safety, HSYA generally possesses low oral bioavailability probably caused by its low membrane permeability, which decreases the effective concentration *in vivo* (Sajid et al., 2021). Since many challenges still exist to limit HSYA clinical application, a large number of studies have been conducted to explore the improvement of chemical instability and bioavailability. Self-double-emulsifying drug delivery system (SDEDDS) has been used to enhance the oral absorption of HSYA and verified no significant toxicity *in vitro* and *in vivo* (Lv et al., 2012). HSYA solid lipid nanoparticles prepared by a warm microemulsion process using approved drug excipients for oral delivery has been demonstrated to increase the oral absorption of HSYA with little cytotoxicity (Zhao et al., 2018). Generally speaking, studies in synthetic biology and metabolic engineering should be further conducted to help improve efficacy, stability, bioavailability and pharmacokinetic properties for accelerating HSYA clinical application.

Since *Carthami flos* is a common part of preparation used in traditional Chinese medicine (TCM), it is necessary to further carry out the compatibility research of HSYA. For example, HSYA and Danshensu achieve synergistic protective effects on cerebral I/R injury through anti-inflammatory and oxidative pathways (Xu et al., 2017). HSYA and astragaloside IV could decrease blood viscosity, plasma viscosity, and attenuate oxidative stress in MCAO rats (Cao et al., 2014). On the other hand, combinations with existing western medication may provide new therapy option. It is reported that HSYA together with acetylglutamine attenuated inflammation and apoptosis process following brain ischemia, and the combination of two drugs exhibited a synergetic effect (Deng et al., 2018). However, it should be pointed out that HSYA can either inhibit or induce activities of CYP1A2, CYP2C11 and CYP3A1 (Xu et al., 2014). Comedication of HSYA with drugs metabolized by CYP1A2 and CYP2C11 will probably result in herb-drug interactions. Hence, more advanced clinical studies should be conducted for fully assessing the safety of HSYA and exploring new compound formulas with HSYA, which may bring benefits not only to brain ischemia patients but also TCM modernization as well.

## 8 Conclusion

In the present review, we summarized the materials about HSYA, including sources, physicochemical properties, safety, protective effects against cerebral I/R and molecular mechanisms *in vivo* (Table 1) and *in vitro* (Table 2). As a natural compound extracted from Chinese herbal, HSYA exerts extensive pharmacological effects in ischemic stroke treatment, including inhibiting excitotoxicity, ameliorating oxidative stress, suppressing inflammation and apoptosis, modulating BBB permeability and regulating autophagy. HSYA suppresses the overactivation of NMDARs following cerebral I/R injury and inhibits excessive release of neurotransmitters, neuronal membrane depolarization, overload of calcium and ischemic LTP mediated by NMDAR to ameliorate excitotoxicity. On the other hand, HSYA increases SOD activity and decrease ROS generation for suppressing oxidative stress, accompanied by attenuating mPTP opening. Apart from that, HSYA has been found to limit ferroptosis and parthanatos for protecting cells from oxidative stress. In addition, HSYA exerts anti-inflammatory effects under cerebral I/R circumstance mainly through activating TLR4-mediated pathway. Moreover, attenuated apoptosis by enhancement of mitochondrial function and improved BBB leakage *via* tight junction regulation also contribute to HSYA-mediated neuroprotective effect against brain ischemic insult. Finally, HSYA ameliorates cerebral I/R injury by regulating autophagy signaling pathway. Therefore, as a potential therapeutic agent for HSYA with a significant application prospect, further study is necessary to be carried out by adopting advanced technologies and methods.

**TABLE 1:** Protective effects of HSYA against cerebral I/R injury under *in vivo* models.

Model	Treatment dosage and duration	Effects	Mechanisms	References
MCAO-induced cerebral I/R injury in rats	2 mg/kg, 4 mg/kg, 8 mg/kg, i.v., -	Reduced infarct volume, improved neurological scores	Alleviation of oxidative stress by decreasing MDA and increasing SOD activity	Wei et al. (2005)
Ca^2+^- and H_2_O_2_-induced insult in rat brain mitochondria	5 μmol/L–80 μmol/L, for 10 min	Inhibited mitochondria swelling	Decreasing ROS generation, enhancing ATP levels and the respiratory control ratio	Tian et al. (2008)
MCAO-induced cerebral I/R injury in rats	25 mg/kg, 50 mg/kg, p.o., for 3 days	Reduced infarct volume, improved brain edema and neurological function	Alleviating inflammatory response by reducing production of TNF-α, IL-1β	Ye and Gao, (2008)
MCAO-induced cerebral I/R injury in rats	1 mg/kg, 5 mg/kg, 10 mg/kg, i.v., -	Reduced infarct volume, BBB leakage and brain edema	Suppressing 12/15-LOX level, inhibiting protein oxidation/nitration	Sun et al. (2012)
MCAO-induced cerebral I/R injury in rats	2 mg/kg, 4 mg/kg, 8 mg/kg, i.v., -	Reduced apoptosis	Increasing Bcl-2 and attenuating Bax, regulating PI3K/Akt/GSK3β pathway	Chen et al. (2013b)
MCAO-induced cerebral I/R injury in rats	2 mg/kg, i.v., -	Reduced infarct volume, improved neurological functions	Promoting autophagy by activating AKT-dependent pathway	Qi et al. (2014)
MCAO-induced cerebral I/R injury in mice	2 mg/kg, i.v., for 4 days	Reduced infarct volume, reduced histopathologic damage of the brain	Reducing pro-inflammatory cytokines through inhibiting TLR4 and suppressing the activation of the NF-κB and MAPK pathways	Lv et al. (2015)
MCAO-induced cerebral I/R injury in rats	8 mg/kg, i.v., -	Reduced infarct volume, improved neurological function	Decreasing TNF-α, inhibiting mPTP opening	Ramagiri and Taliyan, (2016)
OGD-induced cerebral I/R injury in mouse hippocampal slices	0.1 μM–100 μM, -	Decreased excitotoxic damage	Inhibiting postsynaptic NMDAR activity, suppressing pre-synaptic glutamate release, membrane depolarization, NMDAR-dependent iLTP	Wang et al. (2016)
MCAO-induced cerebral I/R injury in rats	5 mg/kg, 10 mg/kg, 20 mg/kg, i.p., for 7 days	Reduced infarct volume, improved neurological scores	Upregulating glucose metabolism, attenuating apoptosis and inflammation process *via* upregulating GFAP and inhibiting the levels of ICAM-1 and inflammatory mediators	Deng et al. (2018)
MCAO-induced cerebral I/R injury in C57BL/6 mice	1 mg/kg, 2 mg/kg, 4 mg/kg, i.p., -	Decreased BBB leakage	Improving tight junction, inhibiting inflammatory reaction *via* TLR4/PI3K/AKT/JNK1/2/14-3-3ε/NF-κB p65 pathway	Lv and Fu, (2018)
MCAO-induced cerebral I/R injury in C57BL/6 mice	5 mg/kg or 20 mg/kg, i.p., for 3 days	Reduced infarct volume	Reprograming the metabolism of phenylalanine	Chen et al. (2019)
MCAO-induced cerebral I/R injury in rats	2 mg/kg, 4 mg/kg, 8 mg/kg, i.v., for 3 days	Reduced infarct volume, improved neurological scores	Elevating GSK3 phosphorylation levels and inhibiting NF-κB activation and iNOS production	Yang et al. (2020)
MCAO-induced cerebral I/R injury in rats	2 mg/kg, 4 mg/kg, 8 mg/kg, i.v., for 3 days	Reduced infarct volume; improved neurological function	Reducing cell apoptosis through regulating SIRT1/FOXO1 pathway	Fangma et al. (2021)
MCAO-induced cerebral I/R injury in rats	5 mg/kg, i.v., -	Reduced infarct volume, improved neurological scores	Reducing ROS, Cyto release, production of ATP, and mPTP opening, suppressing MEK/ERK/CypD pathway	Huang et al. (2021)
Photothrombotic stroke model in C57BL/6J mice	50 mg/kg, i.p., for 3 days	Reduced infarct volume, decreased BBB leakage	Increasing CD31, ZO-1, reducing microglia infiltration and ROS production	Li et al. (2022)
MCAO-induced cerebral I/R injury in rats	-, for 3 days	Reduced infarct volume, alleviate neurobehavioral deficits	Inhibiting autophagy by suppressing HIF-1, BNIP3 and Notch 1	Zhang et al. (2022)

**TABLE 2:** Protective effects of HSYA against cerebral I/R injury under *in vitro* models.

Model	Treatment dosage and duration	Effects	Mechanisms	References
OGD-induced injury in primary cortical cortex neurons	8.2 μM and 24.5 μM, for 24 h	Improved cell viability, protected the neuronal morphology	Decreasing LDH and NO levels	Ye and Gao, (2008)
NMDA-induced injury in rat primary neurons	1 μM and 10 μM, from 1 h before up to 24 h after NMDA insult	Improved the cell survival, decreased apoptosis	Increasing Bcl-2 and inhibiting Bax, down-regulating NR2B-containing NMDA receptors	Yang et al. (2010)
OGD/R-induced injury in PC12 cells	1 μmol/L, 10 μmol/L, and 100 μmol/L, 30 min before OGD and throughout the OGD reperfusion	Improved cell survival, decreased apoptosis	Reducing MDA and increasing SOD, inhibiting the release of cytochrome c, suppressing caspase-3 signaling	Fan et al. (2011a)
OGD/R-induced injury in BV2 microglia	20 μM, 40 μM, 80 μM, 160 μM, 320 μM, 640 μM, and 1,280 μM, for 12 h	Improved cell viability	Attenuating pro-inflammatory factors by inhibiting MAPK/P38 pathway	Li et al. (2013)
LPS-induced injury in microglia and neurons	12 μM, 25 μM, 50 μM, 100 μM, 200 μM, 400 μM, and 800 μM, for 24 h	Inhibited the LPS-induced morphological changes	Attenuating pro-inflammatory factors by inhibiting TLR4-mediated signaling pathway	Lv et al. (2016)
NMDAR-mediated and OGD-induced injury in hippocampal neurons	1 μM, 10 μM, and 10 μM, -	Attenuated excitotoxic neuronal death	Reducing NMDAR-mediated Ca^2+^ influx, stabilizing mitochondrial structures	Wang et al. (2016)
OGD/R-induced injury in BMECs	20 μM, 40 μM, and 80 μM, 2 h prior to OGD/R	Increased the permeability of monolayer BMECs and inhibited BMECs apoptosis	Inhibiting autophagic cell death *via* the activation of Class I PI3K/Akt/mTOR pathway	Yang et al. (2018)
OGD/R-induced injury in primary mouse neurons and PC12 cells	1 μM or 10 μM, for 20 h	Inhibited neuronal cell apoptosis	Inhibiting the expression of phenylalanine, promoting mitochondria function and biogenesis	Chen et al. (2019)
OGD/R-induced injury in BMECs	100 μg/ml, for 12 h after OGD	Improved the cell survival and proliferation, decreased apoptosis	Attenuating intracellular Ca^2+^ concentration, activating antioxidant signaling, decreasing caveolin-1, regulating PHLPP-1/Akt pathway	Cao et al. (2020)
OGD/R-induced injury in primary neuronal cells	160 μM, -	Decreased apoptosis	Down-regulating caspase-3 pathway	Yang et al. (2020)
OGD/R-induced injury in primary hippocampal neurons	40 μM, 60 μM, and 80 μM, during the period of OGD/R injury	Improved cell survival, decreased apoptosis	Increasing GSH-Px, SOD, decreasing ROS, MDA, LDH, activating SIRT1 pathway	Fangma et al. (2021)
OGD/R-induced injury in BMECs	80 μM, 30 min before OGD	Improved cell viability, decreased apoptosis	Decreasing mPTP open *via* regulating MEK/ERK/CypD pathway	Huang et al. (2021)
OGD/R-induced injury in PC12 cells	The cells were treated with HSYA, -	Improved cell survival, decreased apoptosis	Up-regulating cystine/glutamate antiporter system x_c_^−^ and GPX4, decreasing GSH/GSSG, ROS, iron ion and lipid peroxidation by limiting ferroptosis and parthanatos	Chen et al. (2022)
LPS-induced injury in BMECs	10 μM, for 16 h	Protected brain microvessels integrity	Increasing CAT, SOD1, GSH/GSSG and protecting ZO-1 *via* blocking HIF-1α induction of NOX2	Li et al. (2022)

The last but not the least, although a growing number of preclinical studies show the neuroprotective potential of HSYA against cerebral I/R injury, only few clinical studies included patients with acute ischemic stroke have been reported (Li L. J. et al., 2015; Hu et al., 2020). The randomized controlled clinical trial (RCT) is regarded as the “gold standard” for evaluating the effectiveness of drugs. To evaluate the efficacy and safety of HSYA in treating ischemic stroke, more high-quality, multi-center, large-sample, randomized double-blind controlled trials are urgently needed.

## Glossary

I/R: ischemia/reperfusion
BI: brain ischemia
tPA: tissue-type plasminogen activator
FDA: Food and Drug Adminstration
HSYA: hydroxysafflor yellow A
MCAO: middle cerebral artery occlusion
CHS: chalcone synthase
CHI: chalcone isomerase
F3Ms: flavonoid 3′-monooxygenase
ANRs: anthocyanidin reductase
MeJA: methyl jasmonate
BMECs: brain microvascular endothelial cells
OGD/R: oxygen and glucose deprivation/reoxygenation
PI3K: phosphoinositide 3-kinase
Akt: protein kinase B
mTOR: mammalian target of rapamycin
NaCN: sodium cyanide
NMDA: N-methyl-d-aspartate
NMDAR: N-methyl-d-aspartate-receptor
LTP: long-term potentiation
SOD: superoxide dismutase
GSH-Px: glutathione peroxidase
ROS: reactive oxygen species
MDA: malondialdehyde
SIRT1: silent information regulator 1
GPX4: glutathione peroxidase 4
PAR: poly (ADP-ribose)
PARP-1: poly (ADP-ribose) polymerase-1
AIF: apoptosis inducing factor
mPTP: mitochondrial permeability transition pore
LPS: lipopolysaccharide
GSK-3: glycogen synthase knase-3
NF-κB: nuclear factor kappa B
GFAP: glial fibrillary acidic protein
ICAM-1: intercellular adhesion molecular 1
IL-1β: interleukin 1β
TNF-α: tumor necrosis factor-α
TLR-4: Toll-linke receptor-4
MEK: mitogen-activated protein kinase kinase
ERK: extracellular signal-regulated kinase
CypD: cyclophilin D
PHLPP1: PH domain leucine-rich repeat protein phosphatase-1
BBB: blood-brain barrier
Lex: Lexiscan
JNK: c-Jun NH2-terminal kinase
ZO-1: zonula occludens-1
HIF-1α: hypoxia-inducible transcription factor-1α
NOXs: NADPH oxidases
Cav-1: caveolin-1
MMP9: matrix metalloproteinase 9
SYI: safflow yellow injection
IPPH: injectable powder of pure HSYA
CYP: cytochrome P450
Cmax: changes of maximum plasma concentration
AUC: area under the plasma concentration versus time curve
SDEDDS: self-double-emulsifying drug delivery system
NIHSS: National Institute of Health Stroke Scale
BI: Barthel Index
TCM: traditional Chinese medicine
RCT: controlled clinical trial.
